# Mutations in *RNU4ATAC* Are Associated With Chilblain‐Like Lesions and Enhanced Type I Interferon Signalling

**DOI:** 10.1002/eji.202451518

**Published:** 2025-05-25

**Authors:** Nic Robertson, Aakash Joshi, Francesca Ritchie, Ina Schim van der Loeff, David Royan, Angela L. Duker, Gillian I. Rice, Michael B. Bober, Sahar Mansour, David I. Campbell, Mary Brennan, Lindsay Brown, Laura Jones, Eleri Williams, Andrew P. Jackson, Yanick J. Crow

**Affiliations:** ^1^ MRC Human Genetics Unit, Institute of Genetics and Cancer University of Edinburgh Edinburgh UK; ^2^ South West Thames Centre for Genomics St George’s, Epsom and St Helier University Hospitals and Health Group London UK; ^3^ Royal Hospital for Children and Young People Edinburgh UK; ^4^ Great North Children's Hospital RVI Hospital Newcastle Upon Tyne UK; ^5^ Nemours Children's Hospital Wilmington Delaware USA; ^6^ Division of Evolution, Infection & Genomic Sciences, School of Biological Sciences, Faculty of Biology, Medicine and Health University of Manchester Manchester UK; ^7^ Cardiovascular and Genomics Research Institute City St George's University of London London UK; ^8^ Imagine Institute Laboratory of Neurogenetics and Neuroinflammation, INSERM UMR 1163 Université Paris Cité Paris France

**Keywords:** interferonopathy, primordial dwarfism, *RNU4ATAC*‐opathy, Roifman syndrome

## Abstract

Mutations in the non‐coding RNA gene *RNU4ATAC* are associated with growth restriction and complications related to antibody deficiency. Here, we report that innate immune dysfunction is a previously unrecognised feature of this disorder. In particular, painful chilblain‐like lesions are common in *RNU4ATAC* patients and are linked to dysregulated type I interferon signalling.

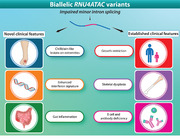

**Abbreviations**
ISG: interferon‐stimulated genes
mRNA: messenger ribonucleic acid
RNA: ribonucleic acid
SD: standard deviations below the mean

To the editor,

Biallelic variants in *RNU4ATAC* cause a spectrum of disorders characterised by growth restriction, microcephaly, skeletal dysplasia and immune deficiency [1]. These phenotypes were historically reported as a series of clinical syndromes, including Roifman syndrome (antibody deficiency with skeletal dysplasia) and microcephalic osteodysplastic primordial dwarfism type I (MOPDI, characterised by extreme growth restriction and brain anomalies). *RNU4ATAC*‐opathy is now the preferred term for all *RNU4ATAC‐*associated disorders [1].

Here, we report four patients with *RNU4ATAC* variants who developed chilblain‐like lesions on their extremities. Similar skin problems can be a feature of dysregulated type I interferon signalling, and, in the two patients assessed, we recorded increased expression of interferon‐stimulated genes (ISGs) in whole blood. We also report a fifth patient with biallelic *RNU4ATAC* mutations who, uniquely, presented with gut inflammation and ulcers on the oropharynx and genitals which responded to immunoglobulin replacement but not steroid treatment. Interferon responses in this patient were normal.

## 1 Case Summaries

**Case 1**: The patient is the second child of a consanguineous couple of Pakistani origin. Microcephaly and growth restriction were noted antenatally, with brain imaging demonstrating absence of the corpus callosum. She was born at 38 weeks gestation by elective caesarean section, with a birth weight of 1.25 kg (−4.6 standard deviations from mean [SD]), length of 37 cm (−6.3 SD) and head circumference (HC) of 26 cm (−5.9 SD). At birth, she was noted to have limb contractures and small digits.

At age 1 year her height and weight were −5 SD with HC −6.7 SD. Immunologically, she had normal‐range IgG but low IgA and IgM. Vaccine responses at 1 year were noted to be suboptimal: the pre‐1 year booster tetanus response was 0.08 IU/mL (normal range >0.1), with pneumococcal IgG titres below 0.35 µg/mL for 3 of the 9 serotypes assayed for which she had received vaccination. Lymphocyte subsets were normal. She started immunoglobulin replacement at 3 years of age after recurrent urinary and upper respiratory tract infections. She has developed hypertension secondary to chronic kidney disease of unknown aetiology and early cataracts. She also has mild to moderate sensorineural hearing loss and eczema.

Sequencing revealed two variants in *RNU4ATAC*, both in homozygosity (n.40C > T and n.65C > T). The n.40C > T variant has been observed in patients with an MOPD1 phenotype, whereas the n.65C > T transition has not previously been associated with disease [2].

**FIGURE 1:**
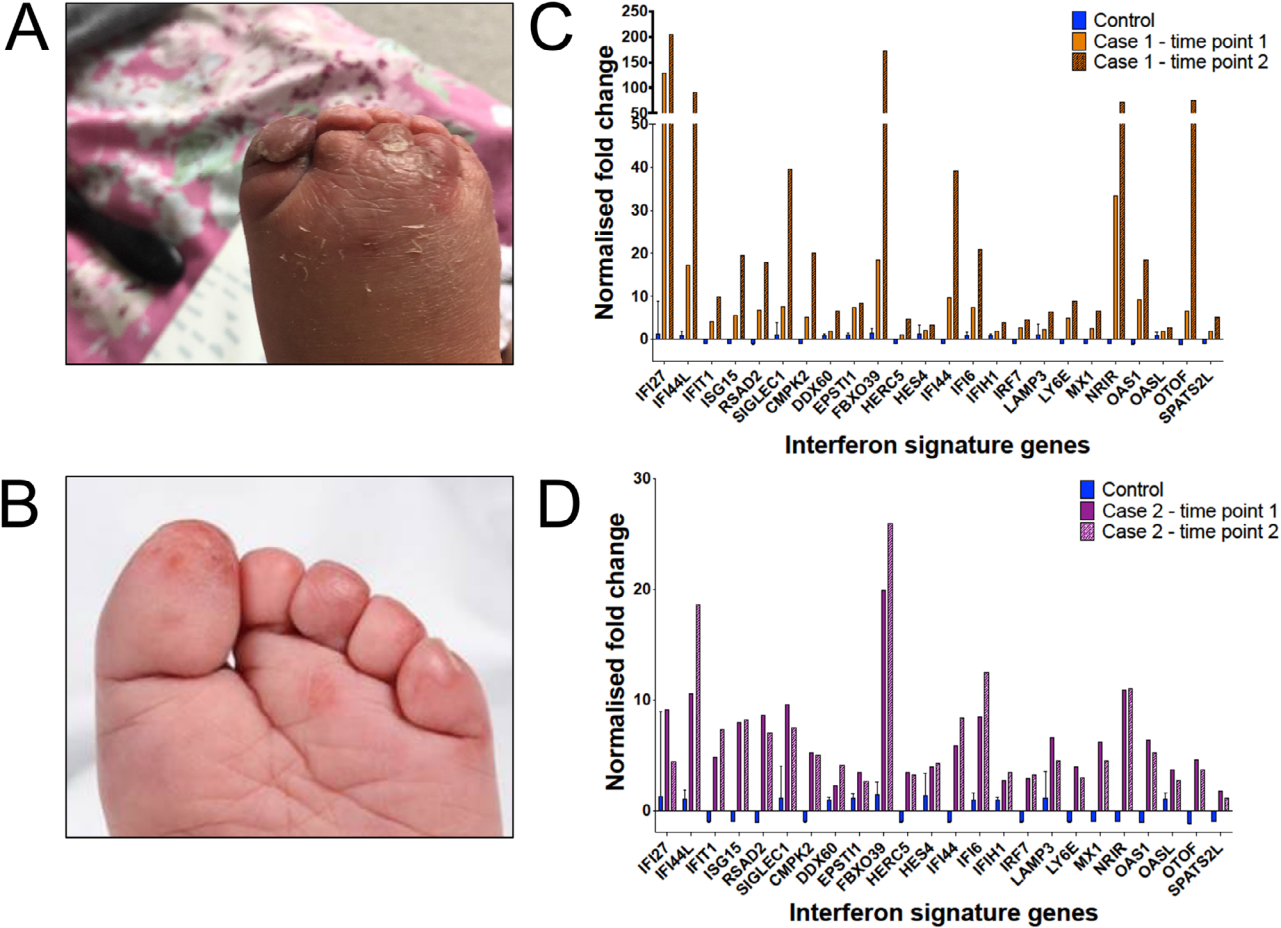
Patients with mutations in *RNU4ATAC* can demonstrate chilblain‐like lesions and an increased interferon signature in peripheral blood. (A) Photograph of dorsum of foot of Case 1, showing marked oedema around lesions on toes. (B) Photograph of foot of Case 2. (C) Interferon‐stimulated gene (ISG) signature in peripheral blood of Case 1. Values are expressed as fold change relative to a previously established reference range (4). (D) Interferon signature of Case 2.

From age 3 years, she developed recurrent chilblain‐like lesions, particularly in the winter months. These are associated with marked oedema and most frequently involve the toes (Figure 1A). The soles of the feet are also often affected. They are not triggered by viral infections and did not occur when she tested positive for Covid‐19 in September 2022. To date, these lesions have responded to treatment with topical steroids. The lesions have not been biopsied, and autoantibody screen was not indicated.

**Case 2**: The second case is a 12‐year‐old male who is the first child of non‐consanguineous parents. Antenatally, he had symmetrical growth restriction, prompting induction of labour at 37 weeks gestation. This was converted to an emergency caesarean section due to foetal heart rate concerns.

He was born with a weight of 1.7 kg (−3 SD) and HC of 29.5 cm (−3 SD). Non‐specific dysmorphic facial features and a ventricular septal defect were noted. He had an initially low red blood cell count which normalised by 3 months of age.

He was treated for possible absence seizures from 5 to 17 months of age, with no recurrence on stopping treatment. Brain imaging at age 6 months showed a focal right periventricular white matter injury but no other abnormalities. He also has hypothyroidism and bilateral macular oedema.

Sequencing showed compound heterozygous variants in *RNU4ATAC*, n.13_15del and n.13C > T. Variants at the n.13 position have previously been described in the context of Roifman syndrome [2]. At 3 months of age, he was assessed for immune deficiency following a urinary tract infection and thrush. He had B cell lymphopenia and hypogammaglobulinemia (low IgG and IgM with undetectable IgA). He commenced immunoglobulin replacement at 4 months of age.

At 4 years of age he started to experience episodes of swollen, red toes which were non‐tender but sometimes associated with peeling of the overlying skin (Figure 1B). Swelling could last up to 2 weeks and was sometimes associated with viral illnesses (two associated with rhinovirus and one with influenza A). An autoantibody screen was negative, and biopsy has not been obtained (Table S1).

## 2 Interferon Responses

Chilblain‐like lesions are a feature of certain type I interferonopathies [3]. Mutations in over 50 genes have been associated with this grouping, including those implicated in the encephalopathy Aicardi–Goutières syndrome (AGS).

Using a previously validated assay with established reference ranges [4], we evaluated the expression in blood of a panel of 24 ISGs in both patients. The assay was repeated at two time points to control for variability (Figure 1C,D). We calculated interferon scores for each patient, taken as the median fold change in ISG expression between the patient and a normalised baseline derived from healthy controls. Case 1 had interferon scores of 5.4 and 14 at the 2 time points, whereas Case 2 had scores of 5.6 and 4.5. All these scores are greater than 2.75, a cut‐off 2 SDs above a mean score calculated from a panel of 29 healthy individuals, and are comparable to scores seen in AGS [4].

## 3 Registry and Literature Review

We reviewed the clinical details of 18 individuals with *RNU4ATAC*‐opathies in the Nemours Primordial Dwarfism Registry and also searched local primordial dwarfism cohorts. In this way, we identified two further patients with *RNU4ATAC* variants and similar recurrent chilblain‐like lesions (Cases 3 and 4; Figure S1 and Supporting Information section). We have not yet been able to assess their interferon signalling status. A literature review identified one further patient with a homozygous n.55G > A transition in *RNU4ATAC* who is described as having chilblain‐like lesions, together with growth restriction and microcephaly [5].

## 4 *RNU4ATAC* Variants Presenting With Gut Inflammation

We also assessed a patient (Case 5) who presented at 15 months of age with intractable diarrhoea together with ulceration of the pharynx, mouth and genital/perianal regions. Endoscopy showed patchy apoptosis and focal inflammation of the colon. Sequencing using an immunodeficiency panel identified disease‐associated variants in *RNU4ATAC* (n.116A > C and n.13C > T; [2]). The ulcers temporarily improved on starting aciclovir and co‐trimoxazole prophylaxis but then relapsed and did not respond to steroids. They resolved after commencing immunoglobulin replacement at 18 months of age. At 2 years, height and HC were −4 and −3 SD, respectively. Full details of his course and treatments are in the Supporting Information section.

Although he did not have ulceration or chilblains on his peripheries, we wondered whether his enteric inflammation was also a manifestation of enhanced interferon responses. ISG testing at a single time point was normal (Figure S2), demonstrating that raised ISGs in blood are not a universal finding in patients with biallelic mutations in *RNU4ATAC*.

## 5 Concluding Remarks

Here, we provide evidence that variants in *RNU4ATAC* can be associated with enhanced type I interferon signalling. We also report, for the first time, gut inflammation and mucosal ulceration as major clinical problems in an *RNU4ATAC* patient. The cases described here expand the spectrum of *RNU4ATAC*‐opathy and suggest that perturbed innate immune signalling is a previously unrecognised feature of this syndrome.

*RNU4ATAC* encodes a non‐coding RNA that forms part of the minor spliceosome, which is responsible for removing minor introns from approximately 700 human mRNA transcripts [1]. The variants in our patients are predicted to disrupt interactions between *RNU4ATAC* and other minor spliceosome components [2]. The n.13 and n.18 variants lie in the Stem 2 structure that forms through base‐pairing with another non‐coding RNA, RNU6ATAC, whereas the n.65 variant is part of a similar interaction in Stem 1. The n.40 and n.116 variants are in protein‐binding domains [2]. Disease‐associated mutations impair minor‐intron excision, but how this leads to disease remains unknown [1].

Mechanistically, enhanced interferon responses in *RNU4ATAC*‐opathy could be triggered either by nucleic acid sensors recognising mis‐spliced transcripts or by altered levels of minor‐intron‐regulated proteins causing dysregulated interferon pathway signalling [3]. In the second case, an endogenous trigger seems likely, given the lesions are more consistently associated with cold weather than viral infections. Understanding the implicated pathways may aid in selecting rational treatments for these distressing lesions.

## Author Contributions

All others identified patients and summarised case notes, apart from Gillian I. Rice who performed ISG assays. Nic Robertson and Yanick J. Crow wrote the manuscript, which was reviewed by all authors.

## Ethics Statement

In the United Kingdom, the study was approved by the Leeds (East) Research Ethics Committee (reference no. 10/H1307/2; Integrated Research Approval System project ID: 62971; Cases 1, 2 and 5) and the Multicentre Research Ethics Committee for Scotland (05/MRE00/74; Case 3). In the United States (Case 4), approval was obtained from the Nemours Institutional Review Board (protocol number 83142).

## Consent

Written informed consent was obtained from all individual participants included in the study. All participants have consented to publication of their data, including (where applicable) anonymous photographs.

## Conflicts of Interest

The authors declare no conflicts of interest.

### Peer Review

The peer review history for this article is available at https://publons.com/publon/10.1002/eji.202451518.

## Supporting information



Supporting Information

## Data Availability

The data that support the findings of this study are available from the corresponding author upon request.
