# Mutations in *RNU4ATAC* are associated with chilblain-like lesions and enhanced type I interferon signalling

**DOI:** 10.1002/eji.202451518

**Published:** 2025-05-01

**Authors:** Nic Robertson, Aakash Joshi, Francesca Ritchie, Ina Schim van der Loeff, David Royan, Angela L Duker, Gillian I Rice, Michael B Bober, Sahar Mansour, David I Campbell, Mary Brennan, Lindsay Brown, Laura Jones, Eleri Williams, Andrew P Jackson, Yanick J Crow

**Affiliations:** 1 MRC Human Genetics Unit, Institute of Genetics and Cancer, University of Edinburgh, Edinburgh, UK; 2 St George’s University Hospitals NHS Foundation Trust, London, UK; 3 Royal Hospital for Children and Young People, Edinburgh, UK; 4 Great North Children’s Hospital, RVI Hospital, Newcastle Upon Tyne, UK; 5 Nemours Children’s Hospital, Wilmington, DE, USA; 6 Division of Evolution & Genomic Sciences, School of Biological Sciences, University of Manchester, Manchester, UK; 7 St Georges University of London, London, UK; 8 Université Paris Cité, Imagine Institute, Laboratory of Neurogenetics and Neuroinflammation, INSERM UMR 1163, Paris, France

**Keywords:** RNU4atac-opathy, interferonopathy, Roifman syndrome, primordial dwarfism

To the editor,

Biallelic variants in *RNU4ATAC* cause a spectrum of disorders characterised by growth restriction, microcephaly, skeletal dysplasia and immune deficiency (1). These phenotypes were historically reported as a series of clinical syndromes, including Roifman syndrome (antibody deficiency with skeletal dysplasia) and microcephalic osteodysplastic primordial dwarfism type I (MOPDI, characterised by extreme growth restriction and brain anomalies). RNU4atac-opathy is now the preferred term for all *RNU4ATAC-*associated disorders (1).

Here, we report four patients with *RNU4ATAC* variants who developed chilblain-like lesions on their extremities. Similar skin problems can be a feature of dysregulated type I interferon signalling, and, in the two patients assessed, we recorded increased expression of interferon stimulated genes (ISGs) in whole blood. We also report a fifth patient with biallelic *RNU4ATAC* mutations who, uniquely, presented with gut inflammation and ulcers on the oropharynx and genitals which responded to immunoglobulin replacement but not steroid treatment. Interferon responses in this patient were normal.

## Case Summaries

**Case 1:** The patient is the second child of a consanguineous couple of Pakistani origin. Microcephaly and growth restriction were noted antenatally, with brain imaging demonstrating absence of the corpus callosum. She was born at 38 weeks gestation by elective caesarean section, with a birth weight of 1.25 kg (-4.6 standard deviations from mean [SD]), length of 37 cm (-6.3 SD) and head circumference (HC) of 26 cm (-5.9 SD). At birth, she was noted to have limb contractures and small digits.

At age 1 year her height and weight were -5 SD with HC -6.7 SD. Immunologically, she had normal range IgG, but low IgA and IgM. Vaccine responses at one year were noted to be suboptimal: the pre-1 year booster tetanus response was 0.08 IU/ml (normal range >0.1), with pneumococcal IgG titres below 0.35 μg/ml for 3 of the 9 serotypes assayed for which she had received vaccination. Lymphocyte subsets were normal. She started immunoglobulin replacement at 3 years of age after recurrent urinary and upper respiratory tract infections. She has developed hypertension secondary to chronic kidney disease of unknown aetiology, and early cataracts. She also has mild to moderate sensorineural hearing loss, and eczema.

Sequencing revealed two variants in *RNU4ATAC*, both in homozygosity (n.40C>T and n.65C>T). The n.40C>T variant has been observed in patients with a MOPD1 phenotype, while the n.65C>T transition has not previously been associated with disease (2).

**Figure 1:**
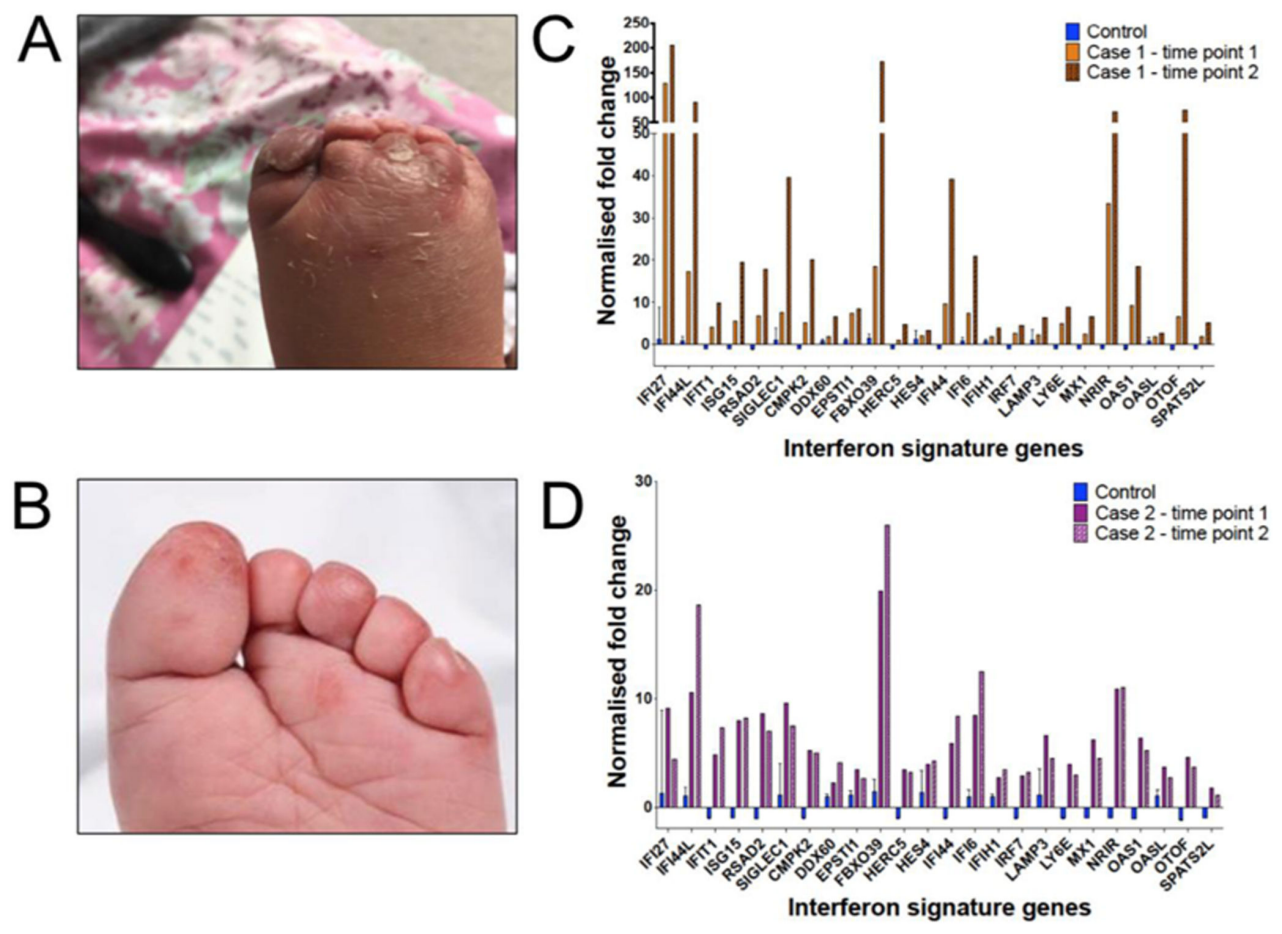
Patients with mutations in RNU4ATAC can demonstrate chilblain-like lesions and an increased interferon signature in peripheral blood. A: Photograph of dorsum of foot of Case 1, showing marked oedema around lesions on toes. B: Photograph of foot of Case 2. C. Interferon stimulated gene (ISG) signature in peripheral blood of Case 1. Values are expressed as fold change relative to a previously established reference range (4). D: Interferon signature of Case 2.

From age 3 years she developed recurrent chilblain-like lesions, particularly in the winter months. These are associated with marked oedema, and most frequently involve the toes (Fig. 1A). The soles of the feet are also often affected. They are not triggered by viral infections, and did not occur when she tested positive for Covid-19 in September 2022. To date, these lesions have responded to treatment with topical steroids. The lesions have not been biopsied, and autoantibody screen was not indicated.

**Case 2:** The second case is a 12-year-old male who is the first child of non-consanguineous parents. Antenatally he had symmetrical growth restriction, prompting induction of labour at 37 weeks gestation. This was converted to an emergency caesarean section due to fetal heart rate concerns.

He was born with a weight of 1.7 kg (-3 SD) and head circumference of 29.5 cm (-3 SD). Non-specific dysmorphic facial features and a ventricular septal defect were noted. He had an initially low red blood cell count which normalised by 3 months of age.

He was treated for possible absence seizures from 5 to 17 months of age, with no recurrence on stopping treatment. Brain imaging at age 6 months showed a focal right periventricular white matter injury but no other abnormalities. He also has hypothyroidism and bilateral macular oedema.

Sequencing showed compound heterozygous variants in *RNU4ATAC*, n.13_15del and n.13C>T. Variants at the n.13 position have previously been described in the context of Roifman syndrome (2). At 3 months of age, he was assessed for immune deficiency following a urinary tract infection and thrush. He had B cell lymphopenia and hypogammglobulinaemia (low IgG and IgM with undetectable IgA). He commenced immunoglobulin replacement at 4 months of age.

At 4 years of age he started to experience episodes of swollen, red toes which were non-tender but sometimes associated with peeling of the overlying skin (Fig. 1B). Swelling could last up to 2 weeks and was sometimes associated with viral illnesses (two associated with rhinovirus, and one with influenza A). An autoantibody screen was negative and biopsy has not been obtained (Table S1).

## Interferon responses

Chilblain-like lesions are a feature of certain type I interferonopathies (3). Mutations in over 50 genes have been associated with this grouping, including those implicated in the encephalopathy Aicardi−Goutières syndrome (AGS).

Using a previously validated assay with established reference ranges (4), we evaluated the expression in blood of a panel of 24 ISGs in both patients. The assay was repeated at two time points to control for variability (Fig. 1C and Fig. 1D). We calculated interferon scores for each patient, taken as the median fold change in ISG expression between the patient and a normalised baseline derived from healthy controls. Case 1 had interferon scores of 5.4 and 14 at the two time points, while Case 2 had scores of 5.6 and 4.5. All these scores are greater than 2.75, a cut-off two standard deviations above a mean score calculated from a panel of 29 healthy individuals, and are comparable to scores seen in AGS (4).

## Registry and literature review

We reviewed the clinical details of 18 individuals with RNU4atac-opathies in the Nemours Primordial Dwarfism Registry, and also searched local primordial dwarfism cohorts. In this way we identified two further patients with RNU4ATAC variants and similar recurrent chilblain-like lesions (Cases 3 and 4; Fig. S1 and Supplemental Information). We have not yet been able to assess their interferon signalling status. A literature review identified one further patient with a homozygous n.55G>A transition in *RNU4ATAC* who is described as having chilblain-like lesions, together with growth restriction and microcephaly (5).

## RNU4ATAC variants presenting with gut inflammation

We also assessed a patient (Case 5) who presented at 15 months of age with intractable diarrhoea together with ulceration of the pharynx, mouth and genital/perianal regions. Endoscopy showed patchy apoptosis and focal inflammation of the colon. Sequencing using an immunodeficiency panel identified disease-associated variants in *RNU4ATAC* (n.116A>C and n.13C>T; ref. 2). The ulcers temporarily improved on starting aciclovir and co-trimoxazole prophylaxis, but then relapsed and did not respond to steroids. They resolved after commencing immunoglobulin replacement at 18 months of age. At 2 years, height and head circumference were -4 and -3 SD, respectively. Full details of his course and treatments are in the Supplemental Information.

Although he did not have ulceration or chilblains on his peripheries, we wondered whether his enteric inflammation was also a manifestation of enhanced interferon responses. ISG testing at a single time point was normal (Fig. S2), demonstrating that raised ISGs in blood are not a universal finding in patients with biallelic mutations in *RNU4ATAC*.

## Concluding remarks

Here, we provide evidence that variants in *RNU4ATAC* can be associated with enhanced type I interferon signalling. We also report, for the first time, gut inflammation and mucosal ulceration as major clinical problems in an *RNU4ATAC* patient. The cases described here expand the spectrum of RNU4atac-opathy, and suggest that perturbed innate immune signalling is a previously unrecognised feature of this syndrome.

*RNU4ATAC* encodes a non-coding RNA that forms part of the minor spliceosome, which is responsible for removing minor introns from approximately 700 human mRNA transcripts (1). The variants in our patients are predicted to disrupt interactions between RNU4ATAC and other minor spliceosome components (2). The n.13 and n.18 variants lie in the Stem 2 structure that forms through base-pairing with another non-coding RNA, RNU6ATAC, while the n.65 variant is part of a similar interaction in Stem 1. The n.40 and n.116 variants are in protein-binding domains (2). Disease-associated mutations impair minor-intron excision, but how this leads to disease remains unknown (1).

Mechanistically, enhanced interferon responses in RNU4ATAC-opathy could be triggered either by nucleic acid sensors recognising mis-spliced transcripts, or by altered levels of minor intron-regulated proteins causing dysregulated interferon pathway signalling (3). In the second case, an endogenous trigger seems likely, given the lesions are more consistently associated with cold weather than viral infections. Understanding the implicated pathways may aid in selecting rational treatments for these distressing lesions.

## Abbreviations

ISG: interferon-stimulated genes
RNA: ribonucleic acid
mRNA: messenger ribonucleic acid
SD: standard deviations below the mean

## Supplementary Material

Supplementary

## Data Availability

The data that support the findings of this study are available from the corresponding author upon reasonable request.
