# Supplementary material for: Mutations in RNU4ATAC are associated with chilblain-like lesions and enhanced type I interferon signalling
Source: Eur J Immunol. Author manuscript; Available in PMC 2025 May 27. (PMC12104551; doi:10.1002/eji.202451518)
Supplement: Supplementary [file EMS205894-supplement-Supplementary.pdf]

Supplemental Information: Mutations in *RNU4ATAC* are associated with chilblain-like lesions and enhanced type I interferon signalling

Robertson *et al* (2025)

Contents:

1. Supplemental Figures 1 and 2
2. Supplemental clinical summaries (Cases 3, 4 and 5)
3. Supplemental Table 1: Summary of cases presented

1. Supplemental Figures

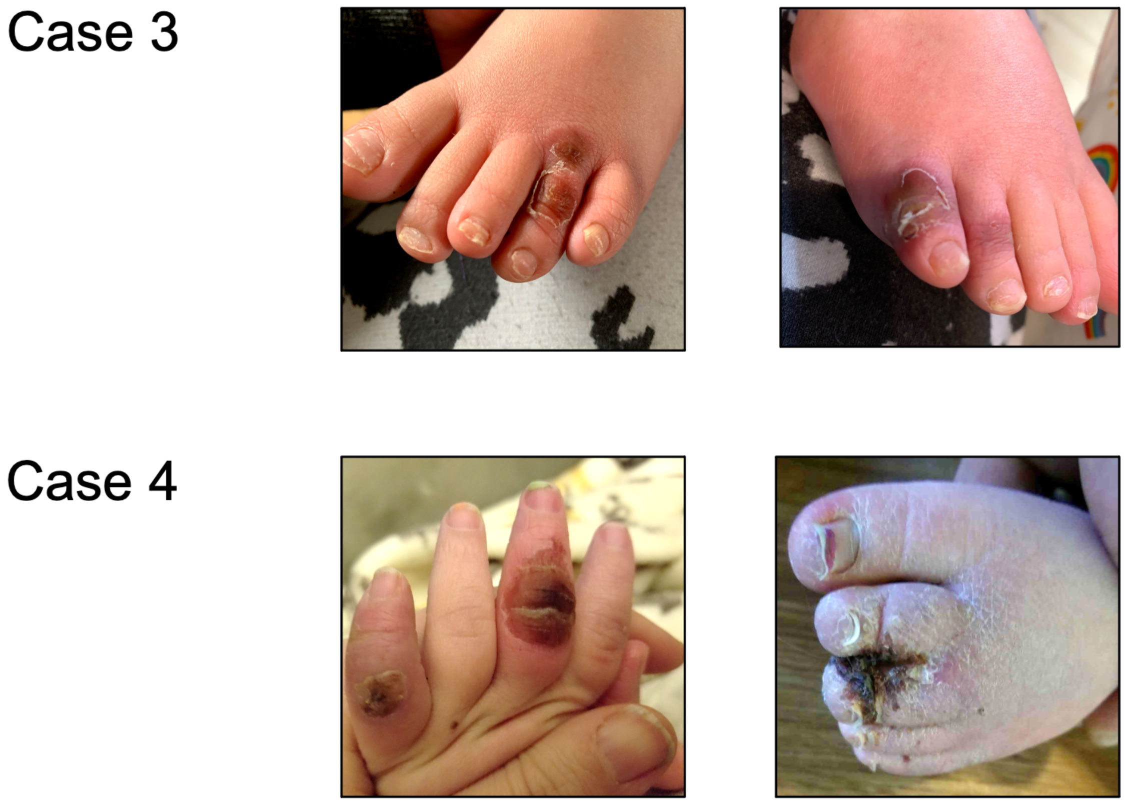

Figure S1: Clinical photographs of hand and foot lesions of Case 3 and Case 4.

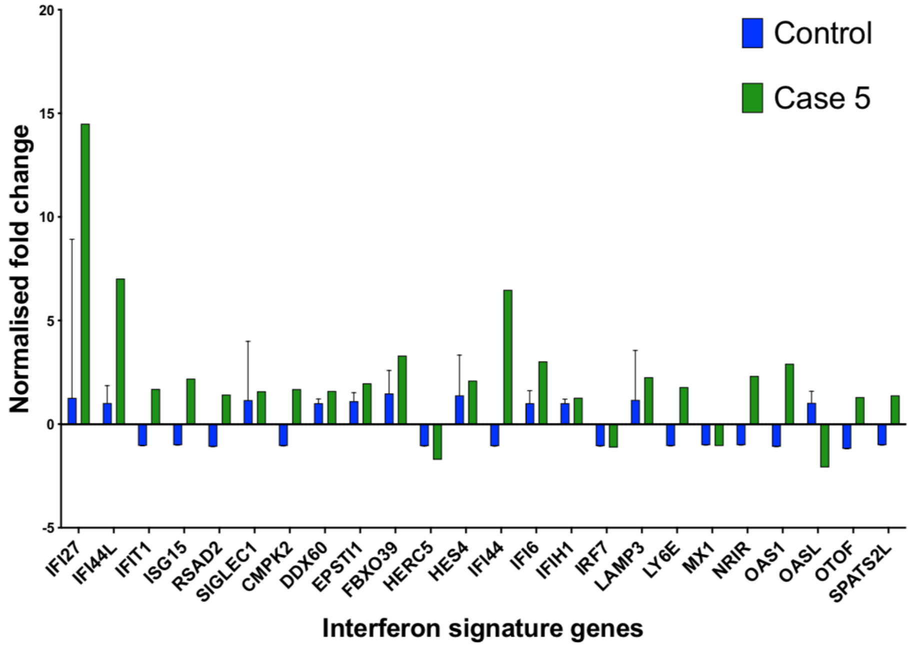

Figure S2: Interferon stimulated gene assay in Case 5. Interferon score is 1.745, indicating no evidence of dysregulated interferon signalling at 1.82 years of age. At the time the sample was taken, the patient was on immunoglobulin replacement but no steroid or other immunosuppressive therapies.

2. Supplemental clinical summaries: Cases 3, 4, and 5

Clinical Summary: Case 3

The third patient is currently 11 years old. He was born by caesarean section at term due to breech presentation. His mother is mixed race British and Gujarati/Pakistani, and his father is white Dutch. He had a birth weight of 1.6 kg (-4.4 SD) and required assistance with feeding and temperature regulation in the neonatal period. Length was 6 SD below the mean at 2 months of age, with head circumference 4 SD below.

He was noted to have subtle features of dysmorphism and motor delay at 5 months of age. A screen for congenital infection was normal. He was further investigated after having apnoeas secondary to viral respiratory infections followed by a persistent cough, and an episode of necrotising pneumonia. An immune screen showed that IgG and IgA titres were both low, with suboptimal vaccine responses. Lymphocyte subsets were normal at 3 years of age, but a year later showed low class-switched memory B cells and ongoing low IgG. He was commenced on immunoglobulin replacement.

Sequencing using a primordial dwarfism panel demonstrated compound heterozygous variants in *RNU4ATAC,* n.13C>T and n.18G>C. The n.18 variant has not been previously reported, but is likely to disrupt Stem II where other disease-associated variants cluster (2).

At 8 years of age he began to experience recurrent discolouration of his fingertips and toes, particularly during the winter months. These lesions are typically oval and inflamed, with a well-demarcated centre surrounded by more diffuse redness (Fig. S1). After becoming manifest, they blister then heal over approximately 3 weeks. They are not associated with clinically-apparent viral infections. Autoantibodies and biopsy have not been indicated.

Clinical Summary: Case 4

This patient is currently 9 years old, and was born in the USA. She has compound heterozygous variants in *RNU4ATAC* (n.116A>C and n.40C>T), both previously reported to be disease-associated (2).

She was born at term, with a birth weight of 2.2 kg (-2.8 SD) and head circumference of 30 cm (-3.7 SD). At birth she was noted to have a short trunk and mesomelia, limb length discrepancy, genu valgum, and pronated pes planus.

Brain MRI identified ectopic pituitary tissue, hypoplastic optic nerves, a simplified gyral pattern and a small corpus callosum. There were concerns about her night vision, and ophthalmologic exam showed axial myopia of her right eye, axial hypermetropia of her left eye, alternating intermittent exotropia and bilateral astigmatism. Head circumference and height were, respectively, 7 SD and 3 SD below the mean at 7 years of age.

Developmentally, she sat at 10 months, crawled at 13 months, pulled to stand at 26 months, cruised at 28 months, and started walking just before 4 years of age. By age 9 years she was fully toilet trained and was starting to read, learn addition and to tell the time. Though her speech is delayed, she can speak in sentences and tell stories. She has learned American Sign Language to communicate with a nonverbal child in her classroom.

Immunologically she has moderately reduced B cell, T cell and NK cell numbers, with normal T-cell proliferation and vaccine responses. She has not required immunoglobulin replacement.

Recurrent colour-changes of her digits began at around 4 months of ag and are her most distressing clinical problem The lesions typically develop over two weeks, becoming increasingly swollen then discharging clear fluid before forming an eschar and gradually healing (Fig. S1). Although they aren't associated with clinically-apparent viral infections, they often occur when other household members are recovering from viral illnesses.

A possible vascular cause was excluded with normal doppler ultrasound and magnetic-resonance angiography. An autoantibody screen was negative (Table S1). Biopsy was planned but, to date, the unpredictable nature of the flares has prevented this.

Clinical Summary: Case 5

This patient is currently 2.5 years old and is the second child of unrelated Caucasian parents. His sibling is four years old and well. Antenatal scans showed short long bones. He was born by elective caesarean at term, with a neonatal course complicated by hypoglycaemia requiring naso-gastric feeding. His birth weight was 2 kg (-3.4 SD), with a

head circumference of 33 cm at 2 weeks of age (-2.7 SD). He was noted to have epicanthic folds, a flat nasal bridge and single palmar creases.

In early life he had several admissions with bronchiolitis, with one episode requiring antibiotics due to consolidation on chest x-ray. He had an echocardiogram which showed an intra-atrial connection.

At 15 months of age he presented with intractable diarrhoea, together with oral, pharyngeal, genital and peri-anal ulceration. The ulcers were painful, and he required opiate analgesia. Viral swabs were negative for herpes-simplex virus, parechovirus and enterovirus. Blood PCRs for CMV, EBV and Adenovirus were negative. Immunologically, he had B cell lymphopenia (undetectable on initial assessment) with normal range CD4 and CD8 cells. Initial immunoglobulins were low-normal range (IgA 1.26, IgG 5.7 and IgM 1.5), but vaccine responses were low and pneumococcal titres did not respond to boosting. Autoantibody and coeliac screens were negative (Table S1).

He commenced naso-gastric feeding and started acyclovir and co-trimoxazole prophylaxis, which was associated with some improvement. Endoscopy showed patchy apoptosis and focal inflammation of the colon. The ulceration then relapsed and he was started on steroid treatment (14 days of budesonide 500 mg twice daily) followed by a prednisolone 2 mg/kg, weaned over a month. He did not show persistent clinical improvement in gut involvement until immunoglobulin replacement was started at 18 months of age.

Sequencing using a trio genome panel showed biallelic variants in *RNU4ATAC,* n.116A>C and n.13C>T, both previously described as disease-associated (2). At 2 years of age, height was 4 SD below the mean and head circumference 3 SD below. His other clinical problems include eczema. Ophthalmic review was normal.

His current treatments are azithromycin prophylaxis (10 mg/kg three times a week) and IV immunoglobulin replacement.

He interferon signalling status in blood was assessed at 21 months of age. The interferon score at a single time-point was 1.745, which is within the normal range (Fig. S2). Of note, some patients with recognised interferonopathies do occasionally have normal interferon signatures (3), so this single negative result does not necessarily exclude dysregulated interferon signalling contributing to his disease.

3. Supplementary Table 1 – Summary of cases presented

Case in manuscript	Country and ID*	RNU4ATAC variants	Growth	Immunology	Autoantibodies	Other problems	Interferon status
Case 1	UK [England] AGS3195	n.40C>T<br>n.65C>T	At 1 year:<br>• Height -5 SD<br>• HC -6.7 SD	• NR IgG, low IgA and IgM<br>• NR LSS<br>• FA: low tetanus and pneumococcus tires<br>• Ig from 3 yr	Not tested	• Severe eczema & multiple food intolerances<br>• Mild/moderate SNHL<br>• CKD & hypertension<br>• Early cataracts<br>• Absent corpus callosum	Clinical chilblains<br><br>Interferon scores: 5.4 and 14 (positive)
Case 2	UK [Scotland] AGS1775	n.13_15 del<br>n.13C>T	At 3 years:<br>• Height -3 SD<br>• HC -3 SD	• Low IgG, IgA and IgM<br>• LSS: B cell lymphopenia<br>• FA not assessed<br>• Ig from 4 mo	Negative results for:<br>• ANA<br>• Anti-dsDNA<br>• ENA (Ro, La, DmDP, U1RNP, Scl-70s, Jo-1, Centromere B)<br>• RF	• Neonatal seizures<br>• VSD<br>• Hypothyroidism<br>• Bilateral macular oedema	Clinical chilblains<br><br>Interferon scores: 5.6 and 4.5 (positive)
Case 3	UK [Scotland] AJ55975	n.13C>T<br>n.18G>C	At 2 months:<br>• Length -6 SD<br>• HC -4 SD	• Low IgG and IgA, NR IgM<br>• LSS: low class-switched memory B cells<br>• FA: low<br>• Ig from 10 yr	• Not tested	• Recurrent viral respiratory infections in early life	Clinical chilblains<br><br>Interferon scores not assessed

Case 4	USA<br>PD128	n.40C>T<br>n.116A>C	At 7 years:<br>• Height -3 SD<br>• HC -7 SD	• Immunoglobulins: low end of normal range<br>• LSS: B cell, NK and CD8 T cell lymphopenia<br>• FA: NR<br>• Ig not required	Negative results for:<br>• ANA<br>• ENA (SS-A / SS-B, Sm, RNP)<br>• ACL Ig M/G<br>• MPO/PR3	• Structural brain anomalies<br>• Eye anomalies<br>• Developmental delay<br>• Eczema	Clinical chilblains<br><br>Interferon scores not assessed
Case 5	UK [England]<br>AGS3807	n.13C>T<br>n.116A>C	At 2 years:<br>• Height -4 SD<br>• HC -3 SD	• Low-normal IgG, IgA and IgM<br>• LSS: B cell lymphopenia<br>• FA: low<br>• Ig from 18 mo	Negative results for:<br>• ANA<br>• Anti-dsDNA<br>• ENA (Ro-52, Ro-60, La, RNP-68, Sm, Scl-70, Jo-1, Centromere, Ribosomal P, chromatin)<br>• Anti-TTG<br>• Anti-mitochondrial<br>• Anti-smooth muscle	• Recurrent viral respiratory infections in early life<br>• Ulceration of oropharynx, genital and peri-anal regions<br>• Colonic inflammation	No clinical chilblains<br><br>Interferon score normal range

*Anonymous ID code linking patient to research databases

Abbreviations:

ACL, anticardiolipin antibodies.
ANA, antinuclear antibody.
Anti-dsDNA, anti double-stranded DNA.
CKD, chronic kidney disease.

ENA , extractable nuclear antigens.
FA, functional antibodies.
HC, head circumference.
Ig, immunoglobulin replacement.
LSS, lymphocyte subsets.
Mo, months.
MPO/PR3, myeloperoxidase/proteinase 3
NR, normal range.
RF, rheumatoid factor
SD, standard deviations below mean.
SNHL, sensorineural hearing loss.
SS-A and SSB, Sjögren's antibodies A and B.
TTG, tissue transglutaminase
VSD, ventricular septal defect.
Yr, years.
